# How can computation advance microbiome research?

**DOI:** 10.1371/journal.pcbi.1005547

**Published:** 2017-09-21

**Authors:** Ruth Nussinov, Jason A. Papin

**Affiliations:** 1 National Cancer Institute, Frederick, Maryland, United States of America; 2 Tel Aviv University, Tel Aviv, Israel; 3 University of Virginia, Charlottesville, Virginia, United States of America

Microbiome science is already in the fast lane, and computations share the credit. How can computations further speed up the pace of microbiome research as it dashes farther, scaling higher summits? What can computations accomplish? What questions can computations help address and what types of algorithms and software should computational biologists aim to develop? These are tantalizing questions that many of us are facing, probably in particular new groups aiming to venture into a relatively nascent and fast-developing area that promises rapid accumulation of data, emergence of alluring new concepts, and significant discoveries. It almost seems like every day, inspiring observations come to light.

Consider, for example, the possible link that was observed between gut bacteria of mice and Parkinson disease, in which changes in the bacteria populating the gut apparently appear to be associated with a decline in motor skills. How can this be? It turns out that 70% of all neurons in the peripheral nervous system are located in the gut, and these are directly connected to the central nervous system through the vagus nerve [1]. A remarkable earlier study suggested that Parkinson disease may start in the stomach, because people who had their vagus nerve cut to treat gastric ulcers exhibited a lower risk of Parkinson disease than those whose treatment involved only a partial dissection [2]. In another striking discovery, it was found that gut bacteria may have a role in autism and, curiously, there is evidence that a single species of gut bacteria can reverse autism-related social behavior in mice [3]. In fact, there is emerging evidence for relationships between disruptions in the human microbiome and cancer [4], cardiovascular disease [5], obesity ([6] and more, e.g., [7]), food allergies [8], and asthma [9], among many other diseases. Beyond these emerging examples, there are well-established links (yet still quite recent!) between the diversity of a healthy gut microbiome and protection against *Clostridium difficile* infection, which has led to therapeutic interventions with remarkable success [10]. The microbiota also has important roles in cancer therapy [11]. Tumor growth can be suppressed by biofilm-producing bacteria [12].

So how can computation accelerate research in the examples above? Broadly, computations can make headway in problems ranging from characterizing taxonomic diversity, classification of microbial species, and tracing their evolution. Computational methods are emerging to facilitate the detection and quantification of diverse patterns among these data, as well as the construction of microbial networks and cross interactions between members of microbial communities. Computation can tackle complex data (e.g., genomic, transcriptomic, proteomic, and metabolomic) on the interactions between microbial communities and their hosts, towards the most challenging question of the quantification of the impact of the human microbiome on our health, as in the examples above.

Beyond the discovery of fundamental biological understanding that computations may enable, the development and applications of efficient algorithms to analyze and sift through data relating to infections and surgical and other medical procedures is yet another complex aspect that may critically affect human health. Uncovering trends in antibiotic resistance may impact our healthcare ecosystem—which computations can help decipher. Computational biology can also develop and apply statistical methods to bacterial communities evolving in different environments in diverse populations to obtain correlations and trends in disparate ecosystems. These and additional emerging data may offer new insights into disease processes and microbiome-inspired therapeutic strategies—insights that may provide leads that can be explored experimentally. To date, next-generation sequencing and computation have already made big strides to elucidate the impact of human genomic variation; for the microbiome, the impact of this variation is yet to be surveyed. Taken together, these may also bear on questions such as how medical approaches influence selection pressure on the human microbiome, which may influence treatment decisions.

New computational approaches are already changing medicine and traditional biology. Computational biologists can spur cutting-edge microbiome research. As the leading journal in computational biology, *PLOS Computational Biology* is planning to catalyze such developments, to stimulate an area that is already surging. In this endeavor, *PLOS Computational Biology* collaborates with other *PLOS* community journals to lead to an upswing in microbiome research for the good of the community. Primary among these is *PLOS Pathogens*. We invite submissions of diverse manuscripts, research, methods, and those describing new and powerful software tools. Together, we look forward to making a difference.
